# Ultrastructure of setae of a planktonic diatom, *Chaetoceros coarctatus*

**DOI:** 10.1038/s41598-022-11484-2

**Published:** 2022-05-09

**Authors:** Yuka Owari, Fumi Nakamura, Yuya Oaki, Hiroyuki Tsuda, Shinji Shimode, Hiroaki Imai

**Affiliations:** 1 School of Integrated Design Engineering, Faculty of Science and Technology, Keio University, 3-14-1 Hiyoshi, Kohoku-ku, Yokohama, 223-8522 Japan; 2 Manazuru Marine Center for Environmental Research and Education, Graduate School of Environment and Information Sciences, Yokohama National University, 61 Iwa, Manazuru, 259-0202 Japan

**Keywords:** Structural biology, Materials science

## Abstract

Silica frustules of most planktonic diatoms have many shallow holes in which the length (L) is smaller than the width (W). The present study focuses on a silica ultrastructure of setae of a planktonic diatom having deep (L/W > 1) holes. Here, we characterized microscopically patterned nanoholes on the silica walls of thick, robust, and hollow setae of a colony of *Chaetoceros coarctatus*. Basically, tetragonal poroid arrangements with and without a costa pattern are observed on the inner and outer surfaces, respectively, for three kinds of curving hollow setae attached to the anterior, intercalary, and posterior parts of the colony. The seta structures including specific poroid arrangements and continuity of deep nanoholes depend on the location. The deep nanoholes ∼90 nm wide are elongated from 150 to 1500 nm (L/W ∼17) with an increase in the wall thickness of the polygonal tubes of the setae. The inside poroid array, with a period of 190 nm in the extension direction of setae, is lined by parallel plates of the costae. However, the poroid arrangement on the outer surface is disordered, with several holes obstructed with increasing wall thickness of the posterior terminal setae. According to the movement of a colony in a fluid microchannel, the thick curving terminal setae is suggested to involve attitude control and mechanical protection. Using an optical simulation, the patterned deep through-holes on the intercalary setae were suggested to contribute anti-reflection of blue light in the wavelength range of 400 to 500 nm for the promotion of photosynthesis in seawater.

## Introduction

Diatoms are single-celled algae that produce intricately structured cell walls made of nanoscopically patterned amorphous silica (SiO_2_)^1^. An individual silica frustule is a cell wall consisting of two valves held together by girdle bands^2,3^. The structures, morphogenetic mechanism, and functions of diatom silica frustules have been studied by many researchers^4–20^. The frustules usually have patterned holes with a width (W) in the range of 100 to 1000 nm^2122^. In most diatom species, the length (L) of holes is generally smaller than their width (W) (Fig. S1 in the Supporting Information (SI)). In this instance, the L/W of the holes ranged from 0.3 to 1 for *Thalassiosira sp.*^21^ and *Coscinodiscus sp*.^22^ The silica wall of *Coscinodiscus* consists of three overlapping porous layers that are composed of nanoparticles 20 to 70 nm in diameter. Various studies have been conducted on the light-trapping effect of silica frustules of diatoms^13,14^. The frustule of *Coscinodiscus centralis* was found to enhance visible-light absorption due to a strong asymmetric property of the pseudo-periodic structures of the silica layer^14^. Holes that are relatively deep (L/W > 1) are suggested to contribute to the functions. Thick silica walls are effective for improving the mechanical property. Deep holes can be utilized for weight saving and mass transfer through the thick wall. However, we rarely observe deep holes in frustules with the exception of *Aulacoseira sp*.^23^.

*Chaetoceros* is one of the most species-rich genera of diatoms in the marine phytoplankton ^24–27^. The cells of *Chaetoceros* forming chains have long setae protruding from each of their four corners^28,29^. Setae, which are much longer than the frustules, are suggested to provide anti-predatory and floating effects^30^. *Chaetoceros* is divided in two subgroups^31^: *Hyalochaete* with thin setae and *Phaeoceros* with thick setae. Commonly, the frustules are not porous and their setae have periodically arranged small pores. The *Hyalochaete* group has relatively thin setae without chloroplasts. Periodically patterned large, shallow holes are linearly arranged on the silica walls of the setae^32^. The *Phaeoceros* group is characterized by rather thick setae that bear chloroplasts. A cell chain of *C. coarctatus* has a pair of posterior and anterior terminal setae and many intercalary ones^33^. The silica architectures of the main bodies and setae are inferred to be essential for mechanical protection and photosynthesis. The ultrastructure of thick setae is interesting because deep nanoholes are arranged in their silica plates. However, dependence of detailed hollow structures of setae including arrangements of deep nanoholes on the location have not been characterized, whereas the mechanism of setae morphogenesis was reported for the subgenus *Phaeocheros*^34,35^. Moreover, experimental evidence has been hardly reported regarding their functions. Thus, we focused on the ultrastructure and function of setae with deep nanoholes of *C. coarctatus* in the *Phaeoceros* group.

In the present study, we characterized the silica frustules of *C. coarctatus*, which has relatively thick and robust setae containing chloroplasts. The micro- and nanoscopic structures, such as polygonal shapes of hollow tubes and patterned deep nanoholes of silica walls, were clarified by detailed observation of various parts of setae. We found patterned nanoholes that penetrate from the inner surface to the outside in the thick wall of three kinds of setae. Moreover, we discuss the functions of curving setae and deep nanoholes according to observations of the movement of a colony in a microchannel and using an optical simulation technique. Finally, our investigation provides further understanding of the morphogenetic process and the relationship between ultrastructures and their functions of various kinds of biosilicas.

## Results and discussion

### Overview on a *C. coarctatus* colony

**Figure 1:**
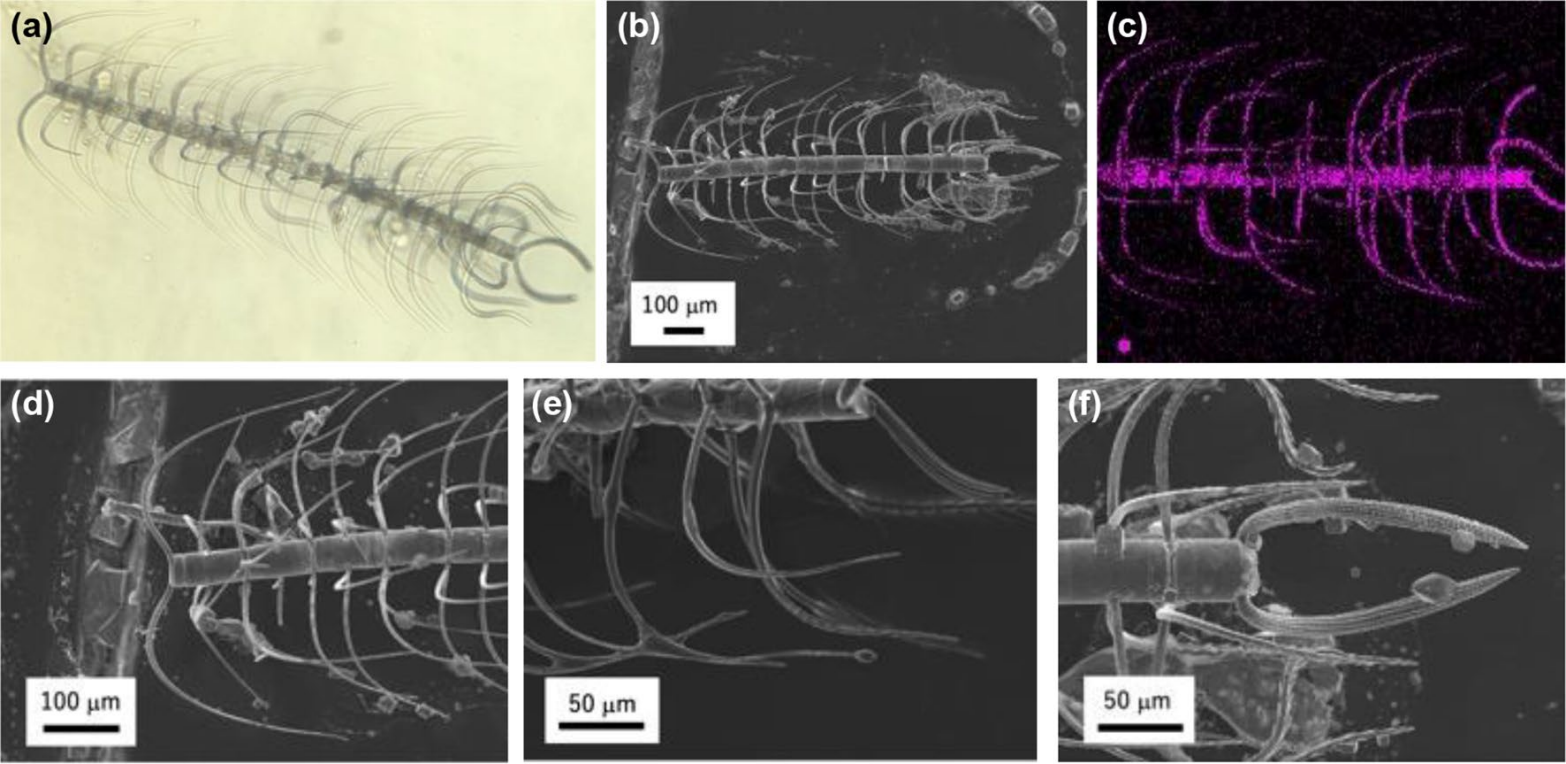
Overall view and enlarged images of a colony of *C. coarctatus*. Optical microscope image (**a**), SEM image (**b**), and elemental mapping of Si by energy-dispersive spectroscopy (EDS) (**c**) of a colony. Enlarged SEM images of M-shaped anterior terminal setae (**d**), intercalary setae (**e**), and U-shaped posterior terminal setae (**f**).

A colony of *C. coarctatus*, ∼1 mm in length, consists of about 15 cells joined in a row (Fig. 1a,b). Four setae, about 5 μm wide and 100 to 300 μm long, are attached on the corners of a frustule of a cell. The setae are classified into three parts: an M-shaped pair of anterior terminal setae, dominant intercalary setae, and a U-shaped pair of posterior terminal setae. As seen in Fig. 1a, most setae (> 90%) are classified as intercalary. All setae curve smoothly and extend posteriorly (Fig. 1d–f). Two setae are joined together at the cell junction (Fig. S2).

**Figure 2:**
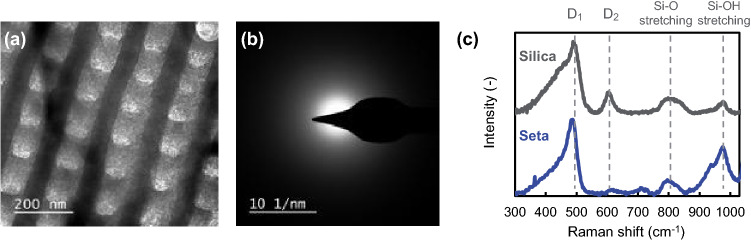
Structural analysis of setae. TEM (**a**) and SAED (**b**) images of a seta wall. Raman spectra of a seta and commercial amorphous silica particles (Reolosil, Tokuyama) (**c**).

From the elemental analysis (Fig. 1c) and a halo pattern in a typical SAED image of a seta wall (Fig. 2a,b), the setae were revealed to be composed of amorphous silica. Figure 2c shows Raman scattering spectra for the biogenic silica and artificial silica nanoparticles. Signals around 486, 619, 795, and 975 cm^-1^ are assigned to a planar four-membered ring (D_1_), a planar three-membered ring (D_2_), a Si–O stretching vibration, and a Si–OH stretching vibration, respectively^36,37^. The positions and intensity ratio of the signal for the setae are consistent with those of silica frustules for other diatom species^38,39^. According to the intensity ratio of the Si–OH stretching vibration band (∼980 cm^-1^) to the Si–O stretching vibration band (∼800 cm^-1^), the biogenic silica contains a higher amount of hydroxy groups than synthetic silica.

### Structures of hollow setae consisting of silica plates with deep nanoholes

We characterized detailed structures of three kinds of setae. As mentioned above, most setae are classified as intercalary. Although hollow shapes consisting of porous plates were reported for various species^28,29^, detailed characterization of the ultrastructures that include deep nanoholes has been insufficient. Here we found that the seta structures including the poroid patterns and continuity of deep nanoholes depend on the location, such as anterior, intercalating, and posterior parts of a colony.

**Figure 3:**
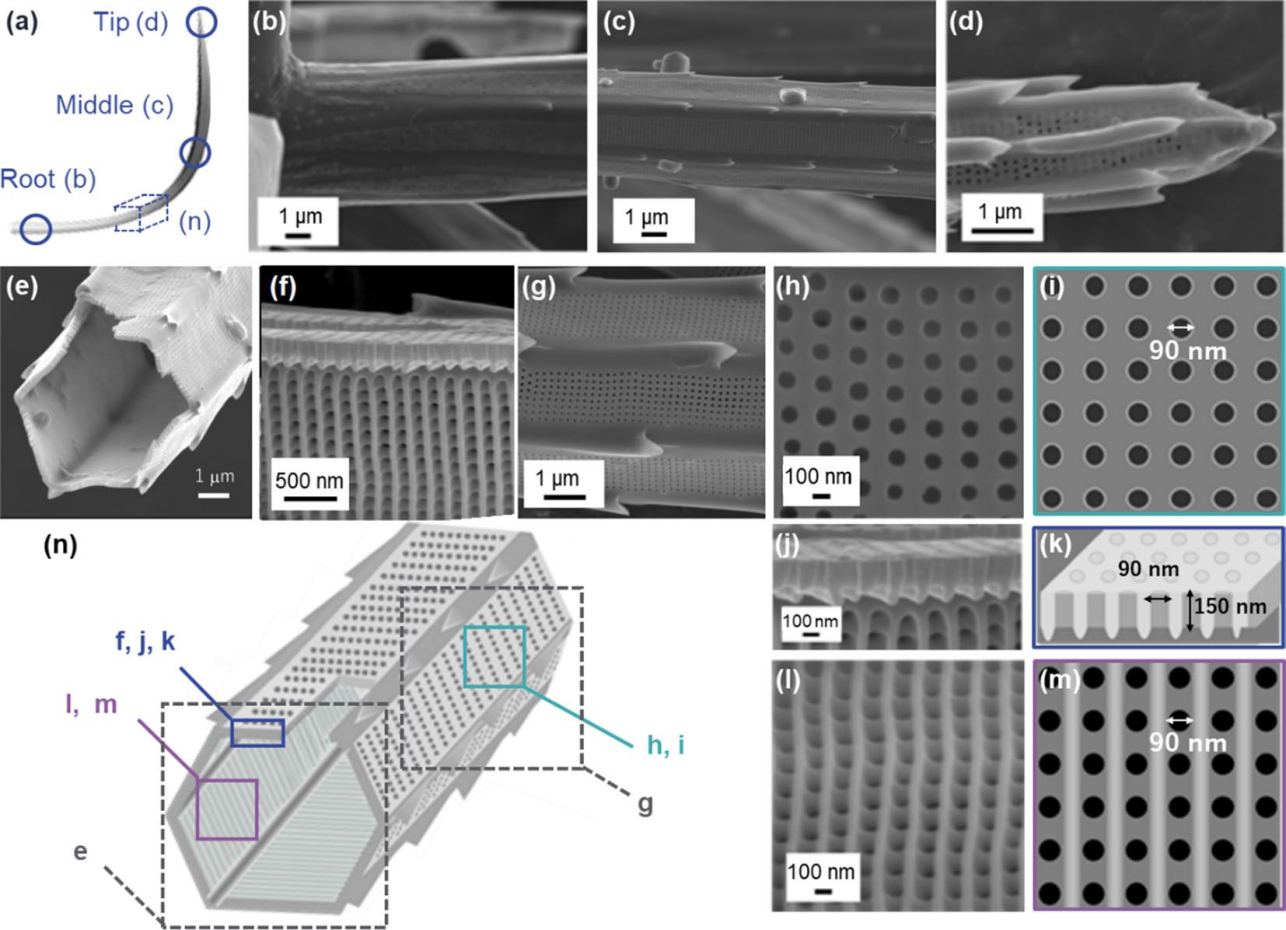
Detailed observation of intercalary setae with SEM images (**b**-**h**, **j**, **l**) and schematic illustrations (**a**, **i**, **k**, **m**, **n**). The appearance of the root (**b**), middle (**c**, **g**), and apex (**d**), the fractured cross section of the middle (**e**, **f**), the poroid arrays on the outer and inner surfaces, and the cross-sectional view of the poroid array (**h**, **j**, **l**).

Curving intercalary setae (Fig. 3a) are cylindrically shaped with a diameter of ∼5 µm at the root near the main frustule (Fig. 3b). The shape of the setae in the intermediate region is a hollow hexagonal prism∼4 µm in diameter with spines on six rims (Fig. 3c,e and Fig. S3a,b in the SI). The apex was found to sharpen with relatively large fin-shaped spines (Fig. 3d). Hollow setae are composed of silica plates ∼150 nm thick and ∼2 µm wide that have periodically patterned poroid arrays (Fig. 3e–g,n). The pores ∼90 nm wide are arranged in a tetragonal pattern with a spatial period of ∼200 nm (Fig. 3h, i). We found the presence of periodical platy projections (costae) ∼100 nm high that are arranged vertically to the extension direction of the setae on the inner surface (Fig. 3j–m). Here, we observed deep nanoholes (L/W > 1) because, from the side-view images, the length was evaluated to be ∼150 nm (Fig. S4 in the SI). The periodicity of the nanoholes seemed to be lined by the costae.

**Figure 4:**
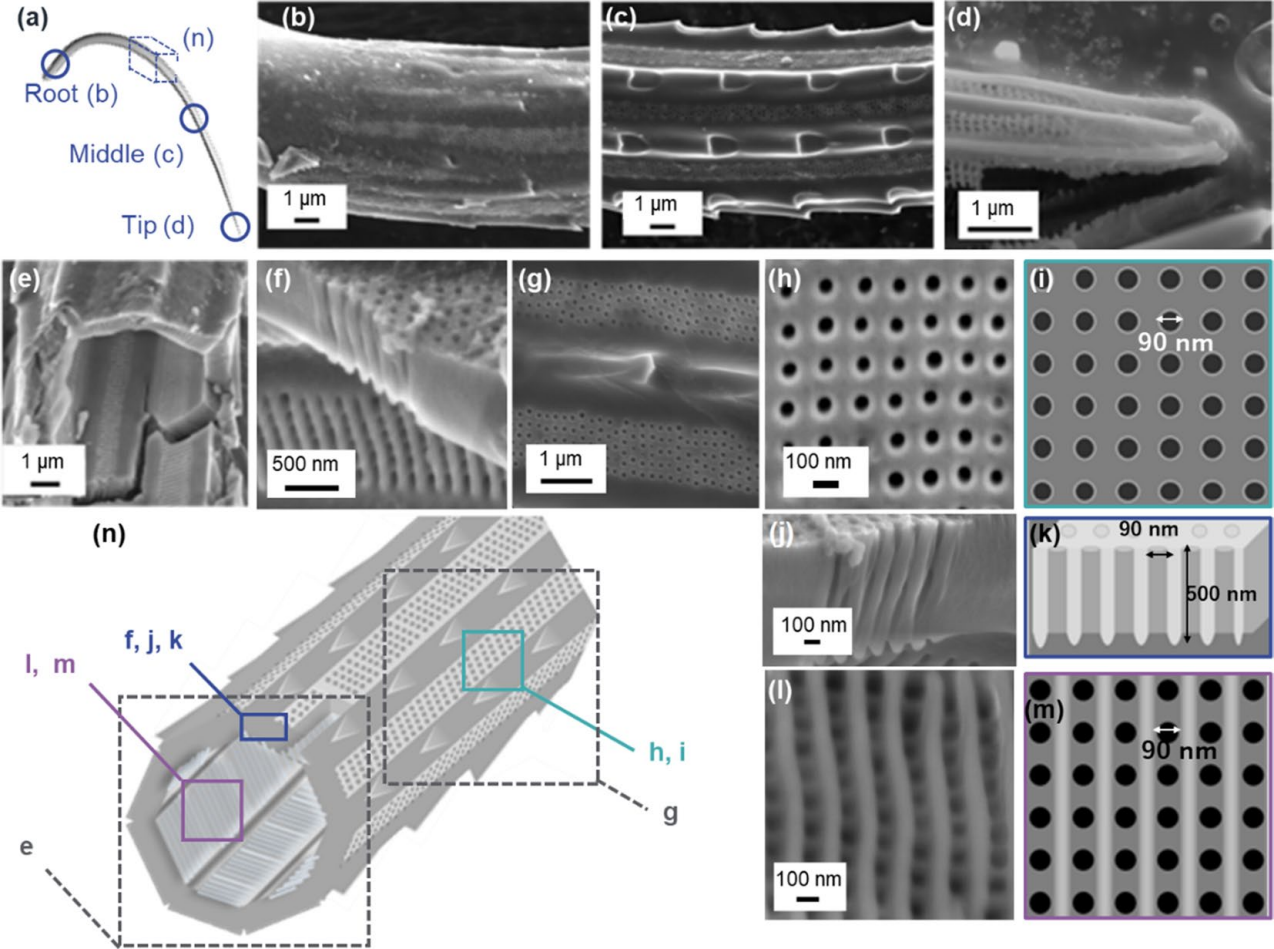
Detailed observation of anterior setae with SEM images (**b**–**h**, **j**, **l**) and schematic illustrations (**a**, **i**, **k**, **m**, **n**). The appearance of the root (**b**), middle (**c**, **g**), and apex (**d**), the fractured cross section of the middle (**e**, **f**), the poroid arrays on the outer and inner surfaces, and the cross-sectional view of the poroid array (**h**, **j**, **l**).

An M-shaped pair of terminal setae are attached at the anterior end of a colony (Fig. 1a,b,d). At the root near the main frustule (Fig. 4a), the setae are cylindrically shaped with a diameter of ∼7 µm (Fig. 4b). The shape of the setae in the intermediate region is a hollow octagonal cylinder∼8 µm in diameter with spines on the rims (Fig. 4c, e and Fig. S3c,d in the SI). The shark-fin-shaped spines were observed to be thick in comparison with those of the intercalary setae. The apex sharpens gradually with relatively large protruding rims (Fig. 4d). Hollow setae are composed of silica plates ∼500 nm thick and ∼1 µm wide that have periodically patterned poroid arrays (Fig. 4e–g, n). Pores ∼90 nm in diameter are arranged in a tetragonal pattern with a spatial period of ∼200 nm (Fig. 4h,i). We found nanoholes in the cross-sectional image of setae because the pores penetrate both surfaces (Fig. 4j,k). The costae ∼100 nm high are arranged vertically to the extension direction of the setae on the inner surface (Fig. 4l,m). Here, we observed very deep nanoholes (L/W > 5) because, from the side-view images, the length was evaluated to be ∼500 nm (Fig. S4 in the SI).

**Figure 5:**
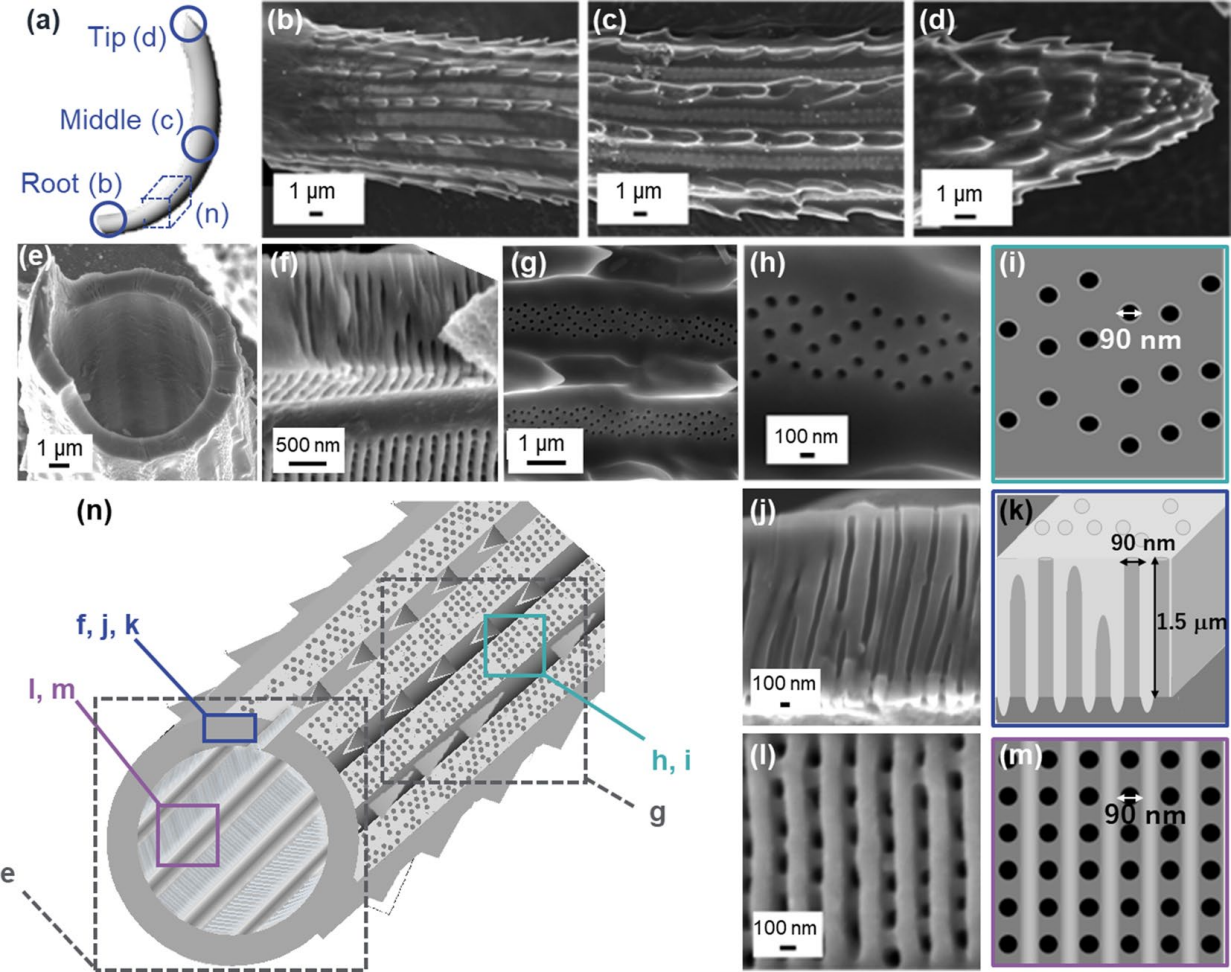
Detailed observation of posterior setae with SEM images (**b**–**h**, **j**, **l**) and schematic illustrations (**a**, **i**, **k**, **m**, **n**). The appearance of the root (**b**), middle (**c**, **g**), and apex (**d**), the fractured cross section of the middle (**e**, **f**), the poroid arrays on the outer and inner surfaces, and the cross-sectional view of the poroid array (**h**, **j**, **l**).

A U-shaped pair of terminal setae are attached at the posterior end of a colony (Fig. 1a,b,f). At the root near the frustule (Fig. 5a), the posterior setae are a cylinder with a diameter of ∼12 µm (Fig. 5b). The setae in the intermediate region are a hollow circular cylinder ∼15 µm in diameter with spines on the rims (Fig. 5c,e and Fig. S3f. in the SI). The shark-fin-shaped spines were observed to be thick in comparison with those of the intercalary setae. The apex sharpens steeply with relatively large spines (Fig. 5d). Hollow setae are composed of silica plates ∼1.5 µm thick and ∼1.5 µm wide that have random arrays of pores ∼90 nm in diameter (Fig. 5e–i, n). On the other hand, we found the presence of costae ∼100 nm high that are arranged with a spatial period of ∼150 nm and vertical to the extension direction of the setae on the inner surface (Fig. 5l,m). The pores are regularly arranged between the costae, while the pores on the outer surface are sparse and random. The nanoholes connecting the inner surface are observed in the cross-sectional image of setae. Here, we observed extremely deep nanoholes (L/W > 15) because, from the side-view images, the length was evaluated to be ∼1.5 µm (Fig. S4in the SI). However, some of the nanoholes are not connected to the outer surface. The arrangement on the outer surface is disordered, with several holes obstructed with increasing wall thickness (Fig. 5j,k).

**Figure 6:**
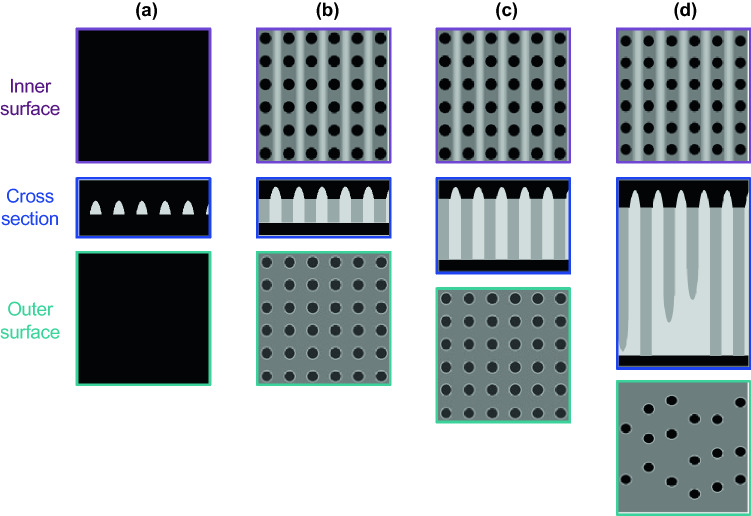
A schematic illustration of the morphogenesis of deep nanoholes with costae with the increasing thickness of a plate. Costae are initially arranged on the inner surface of a silica plate (**a**). The pores are arranged between costae (**b**). The depth of the nanoholes increases with increasing plate thickness (**c**). Several nanoholes collapse with growth of the plate to more than 1000 nm (**d**). Schematic illustration of the poroid arrangements (**b**–**d**) show real structures based on SEM observations. On the other hand, the initial ladder structure consisting of costae (**a**) is presumed from the real poroid structures.

The setae of *C. coarctatus* are featured by periodically patterned deep nanoholes of silica plates composed of hollow prisms. Hollow setae are composed of silica plates periodically patterned with poroid arrays. Figure 6 summarizes the ultrastructure of deep nanoholes of setae. Pores ∼90 nm in diameter are arranged in a tetragonal pattern with a spatial period of ∼200 nm. Costae ∼100 nm high are arranged vertically to the extension direction of the setae on the inner surface. The thicknesses of the silica plates are 150, 500, and 1500 nm for intercalary, anterior, and posterior setae, respectively (Fig. 6b–d). The depth of the nanoholes increases with increasing plate thickness. The periodicity of the poroid arrangements is highly ordered for both the inner and outer surfaces of relatively thin plates. On the other hand, the pores are randomly arranged on the outer surface of the thick plates, although the poroid array is lined by costae on the inner surface. The randomness of the poroid arrangement on the outer surface of the thick plates is ascribed to collapse of the nanoholes on the outer side (Figs. 5j,k and 6d). These facts suggest that the ordered parallel array of costae is initially produced on the inner surface. In a previous study, the morphogenesis mechanism of setae with ladder-shaped silica was examined^40^. Thus, we assume that a ladder structure is the basis for the formation of deep nanoholes of setae (Fig. 6a). The nanoholes are then formed between the costae with increasing plate thickness. When the thickness is less than 500 nm, the nanoholes penetrate to the outer surface of the plate. However, some of the nanoholes collapse with growth of the plate to more than1000 nm.

### Observation of a colony’s movement in a fluid microchannel

**Figure 7:**
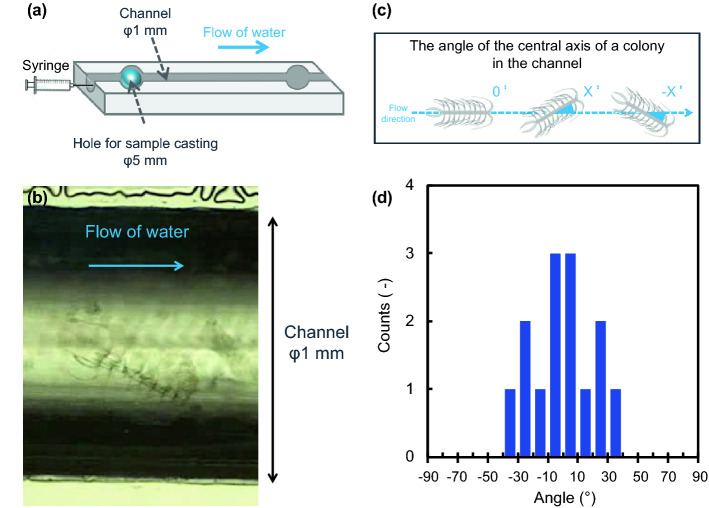
Observations of a dead colony of *C. coarctatus* in a flow of water in a microchannel 1 mm in diameter. Schematic illustration of the flow system including the microchannel (**a**), an optical microscopy image of a colony of *C. coarctatus* in the microchannel (**b**), a schematic illustration of the angle of a colony in the microchannel (**c**), and the distribution of the angle of a colony (**d**).

A subgenus of *Phaeoceros* has a strong and robust setae that has been reported to be capable of mechanically damaging the gills of fish^41,42^. We assume that unidirectionally curving setae of *C. coarctatus* are advantageous for shinnying through the small gap between the gills. Here, we observed the movement of a dead colony in a fluid channel 1 mm in diameter under an optical microscope (Fig. 7a). The flow rate of water was fixed to at about 2 mm/s. The anterior setae was found to be at the head of a colony during flow in the microchannels (Fig. 7b). The angle of the central axis of a colony in the channel was evaluated statistically by repeating the flow experiment (Fig. 7c,d). The colony in a flow was found to be arranged mainly parallel to the channel walls. According to the movement of a colony in a fluid microchannel, the curving of terminal setae is suggested to involve attitude control and mechanical protection. The smooth movement of a colony would be supported by the curving morphology of setae. Thick silica walls with deep nanoholes are needed to strengthen the anterior and posterior setae to protect the colony.

The microscopic dynamic motion in fluid is difference from that on macroscale because of small characteristic length. Thus, the demonstration experiment is fruitful to clarify the real colony motion in a flow channel even if it is obvious that the colony will orient itself to have minimum drag. We successfully demonstrated the motion of the colony that was arranged to be parallel to the channel. We assumed that the attitude control is related to the streamlined shape of setae and the relatively thick posterior setae as an anchor. Moreover, the contact of the anterior setae to the channel wall was observed. This suggests that thick and tough setae are needed to protect the colony in the flow channel that is similar to gill aperture of fish.

### Simulation of optical properties of setae with deep nanoholes

**Figure 8:**
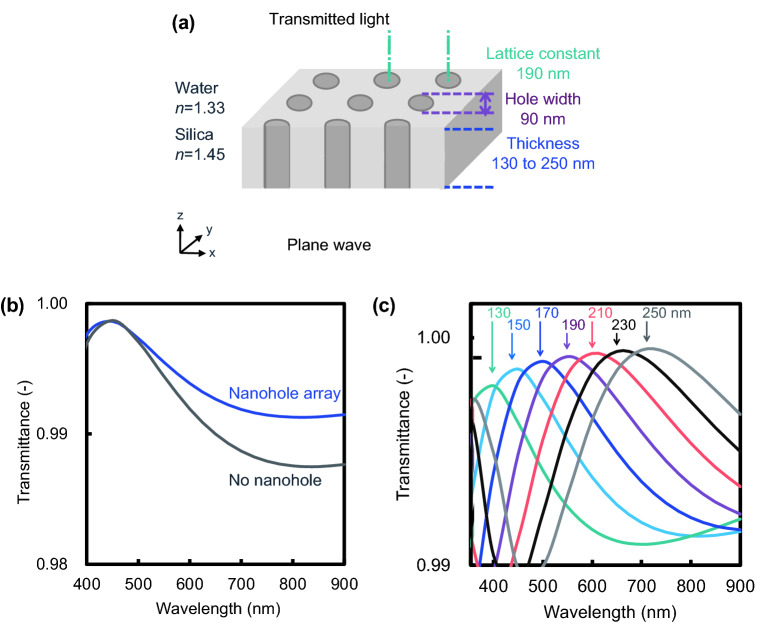
Simulated transmittance spectra of a silica plate with nanoholes using the 3D-FDTD method. A schematic model for 3D-FDTD simulation (**a**). Comparison of transmission spectra with and without nanohole structure; the thickness of the silica plate is 150 nm (**b**).Transmitted spectral variation for different thicknesses of silica plates from 130 to 250 nm (**c**).

Since setae of *C. coarctatus* contain chloroplasts, the characterization of the optical property of the silica shells is important for understanding their biological function. Most setae are classified as intercalary. Thus, we studied the optical property of silica plates with nanoholes 90 nm in diameter with a spatial period of 190 nm in intercalary setae with optical simulation using the 3D finite-difference time-domain (3D-FDTD) method (Fig. 8a).

Figure 8b shows the change in the transmittance of a silica plate 150 nm thick in the visible light region with and without nanoholes. The presence of nanoholes in the silica plate is advantageous for transmittance in visible light. Figure 8c shows the variation of transmittance through a silica plate with a nanohole array in the visible light region with plate thickness changing plate from 130 to 250 nm. The transmission band shifts to a higher wavelength as the thickness increases. For a silica plate thickness of 150 nm, which actually constitutes the *C. coarctatus* intercalary setae, the total transmission peak was at a wavelength around 450 nm. The maximum absorption peak of chloroplasts in diatoms is around 450 nm^43^. The difference between with and without nanoholes in water is not significant for the total amount of incident light. However, visible light penetrates through many silica walls with the nanohole array because a colony has many intercalary setae more than 20. Although the anti-reflection effect with the nanohole array is relatively small in water, the accumulation of the transmission gain would contribute to light acquisition. Thus, the silica plates of intercalary setae of *C. coarctatus* are inferred to be designed to absorb light effectively for photosynthesis.

## Conclusion

Ultrastructures and functions of setae of the planktonic diatom *Chaetoceros coarctatus* were characterized by detailed observation, movement analysis, and optical simulation. Microscopic hollow shapes and nanoscopically patterned deep nanoholes of amorphous silica walls of setae were shown for the anterior, intercalary, and posterior parts of the colony. Tetragonally patterned nanoholes that penetrate from the inner surface to the outside are elongated with an increase in the wall thickness. Patterned nanoholes of thin silica plates for the intercalary setae were suggested to contribute to higher transmittance of blue light, in the range of 400 to 500 nm, in seawater. Anterior and posterior terminal setae composed of thick silica plates would be utilized for mechanical protection. Our findings will lead to the understanding of the morphogenetic process and the relationship between ultrastructures and their functions of various kinds of biosilicas.

## Experimental

Plankton samplings were conducted at 35° 09.45′N, 139° 10.00′E in the western part of Sagami Bay in the south of Japan, on R/V Tachibana of the Manazuru Marine Center for Environmental Research and Education, Yokohama National University. *C. coarctatus* were collected by a plankton net (caliber: 80 cm, side length: 3 m, mesh size: 100 μm)*.*

Living specimens of *C. coarctatus* were sorted from the plankton samples and transferred to a vessel containing pure water for optical microscope observation. *C. coarctatus* were fixed with ethanol, and organic components were removed with a sodium hypochlorite solution. The samples were then dried and coated with osmium for detailed observation using a scanning electron microscope operated (SEM, FEI Helios G4 UX, JEOL JSM-7100) operated at 2.0–15.0 kV. The compositions were identified using Raman scattering spectroscopy and energy-dispersive X-ray spectroscopy (EDS, JEOL JED-2300). Micro-Raman was performed using a laser confocal microscope (inVia, Renishaw). The 532 nm excitation laser was focused on the sample surface with a 100 × objective lens. The size of the laser spot was about 1 μm in diameter. Crystallinity was characterized by transmission electron microscopy (TEM, FEI Tecnai G2). The setae were crushed with a needle and dropped with water on a copper grid. A suspension containing crystals was quickly dried for a few minutes on a copper grid for TEM observation.

We observed the movement of a dead colony in a fluid channel 1 mm in diameter under an optical microscope. We put water containing a colony into the channel using a syringe. The flow rate of water was fixed to at about 2 mm/s. The angle of the central axis of a colony in the channel was evaluated statistically by repeating the flow experiment. The light transmittance of the nanoholes of the setae was simulated using the 3D finite-difference time-domain (3D-FDTD) method. Details of the condition are described in the SI with Fig. S5.

## Supplementary Information

Supplementary Information.
