# Supplementary material for: Ultrastructure of setae of a planktonic diatom, Chaetoceros coarctatus
Source: Sci Rep. 2022 May 9;12:7568. doi: 10.1038/s41598-022-11484-2 (PMC9085750; doi:10.1038/s41598-022-11484-2)
Supplement: Supplementary file 1 — Supplementary Information. [file 41598_2022_11484_MOESM1_ESM.pdf]

Supporting Information

Ultrastructure of setae of a planktonic diatom, *Chaetoceros coarctatus*

Yuka Owari[1] • Yuya Oaki[1] • Fumi Nakamura[1] • Hiroyuki Tsuda[1] • Shinji Shimode[2] • Hiroaki Imai[1]*

Address

1: Department of Applied Chemistry, Faculty of Science and Technology, Keio University, 3-14-1 Hiyoshi, Kohoku-ku, Yokohama 223-8522, Japan /E-mail*: hiroaki@applc.keio.ac.jp

2: Manazuru Marine Center for Environmental Research and Education, Graduate School of Environment and Information Sciences, Yokohama National University, 61 Iwa, Manazuru 259-0202, Japan.

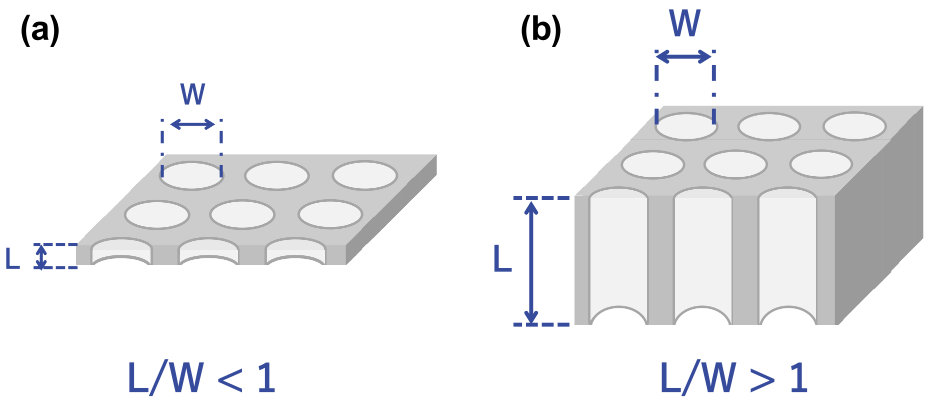

Fig. S1 Schematic diagrams of shallow and deep holes. The definition of shallow holes is the length (L) of holes is generally smaller than their width (W) (a), deep holes is the length (L) of holes is generally larger than their width (W) (b).

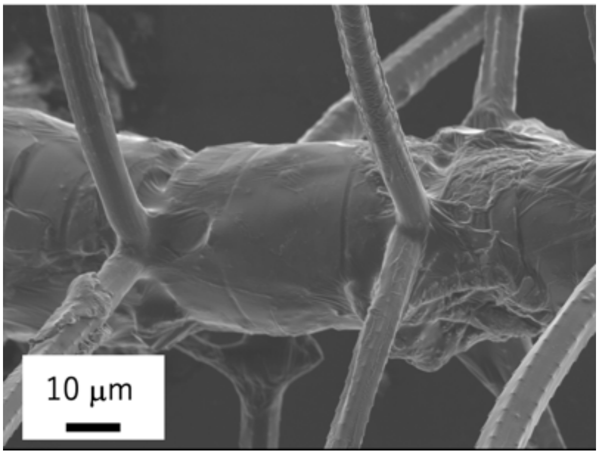

Fig. S2 A SEM image of an intercalary cellular injection of a *C. coarctatus* colony. Four setae extend from the four sides of a cell.

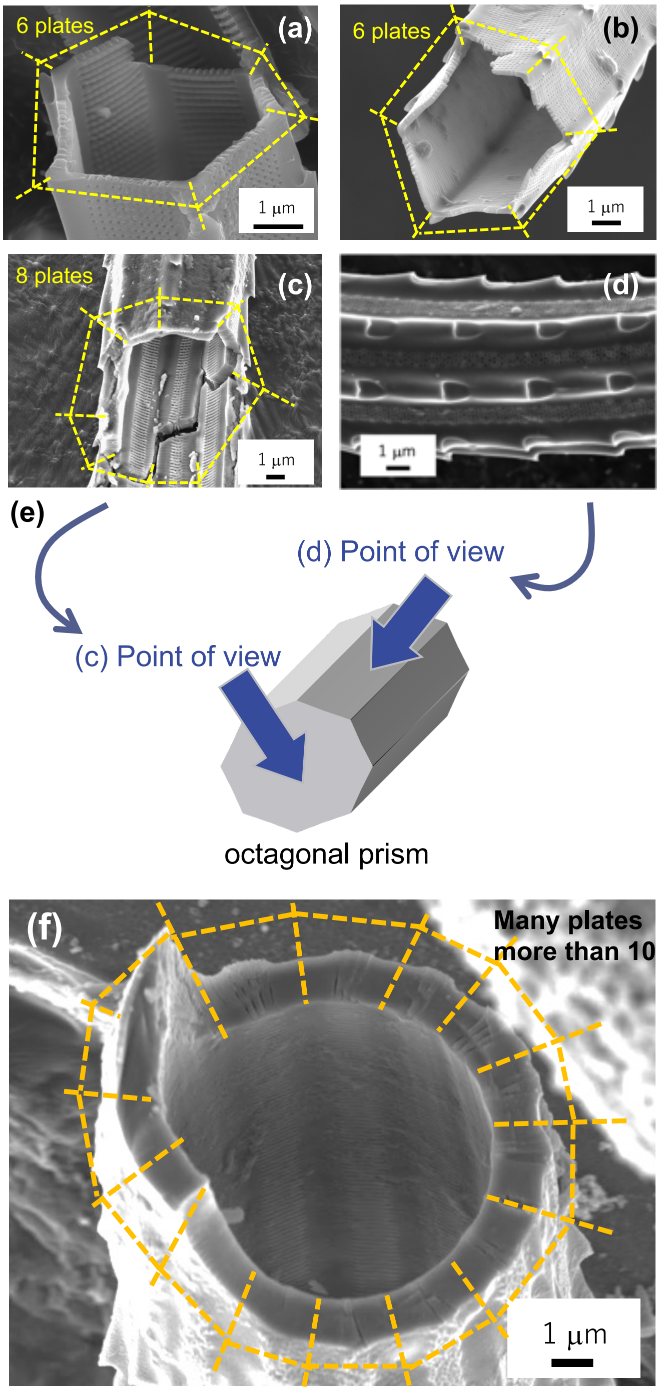

Fig. S3 SEM images of intercalary (a, b), anterior terminal (c, d), and posterior terminal (f) setae in the intermediate region. Schematic diagram (e) shows the point of view of anterior terminal setae. The polygonal shapes of the tubular structures are deduced from the number of silica plates. The shape of the intercalary setae is a hollow hexagonal prism. The anterior terminal setae are a hollow octagonal cylinder. The posterior terminal setae are a hollow circular cylinder consisting of silica plates more than 10.

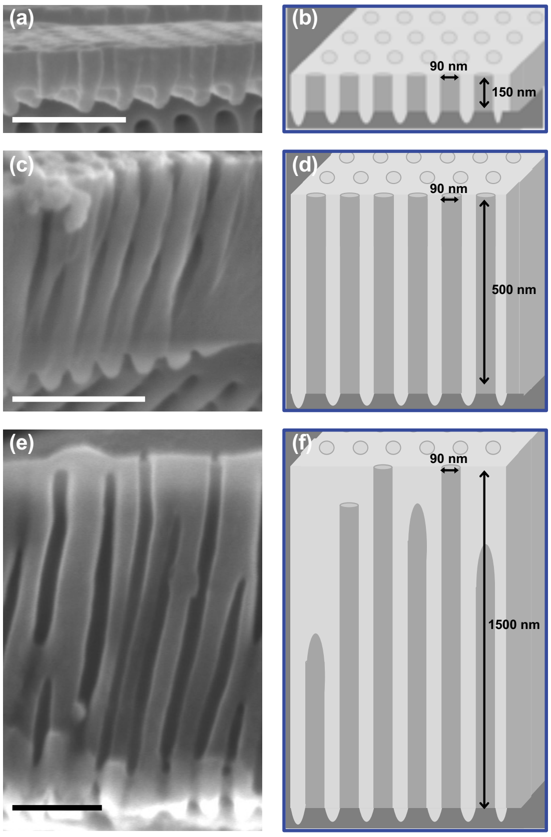

Fig. S4 Detail of nanohole structure of setae cross section. SEM images of intercalary (a), anterior terminal (c), posterior terminal (e) seta cross section. Schematic illustrations of intercalary (b), anterior terminal (d), posterior terminal (f) seta cross section. Scale bar in SEM images are 500 nm.

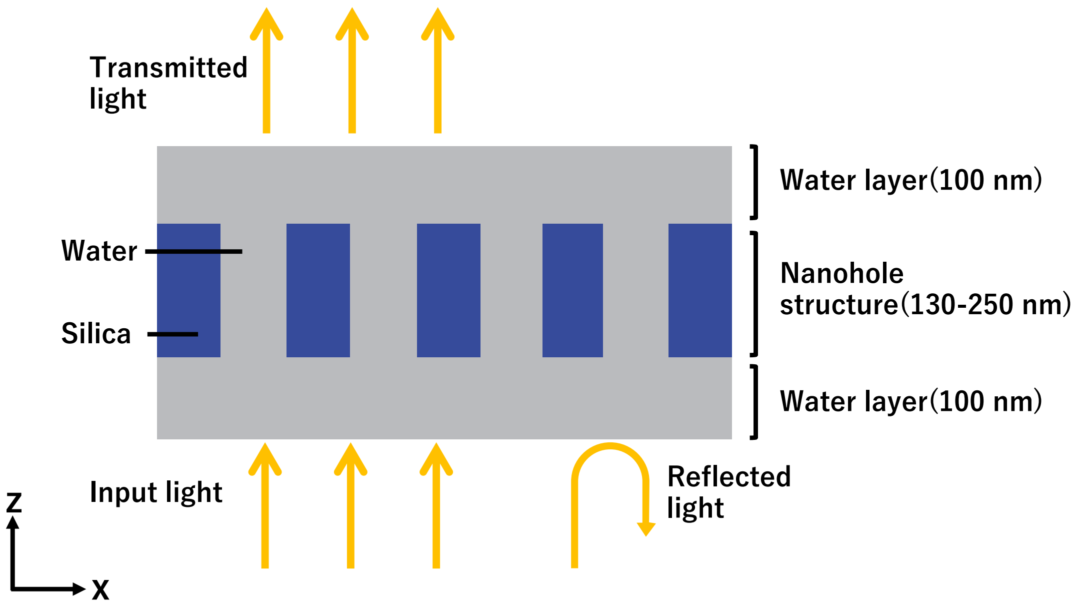

Fig. S5 A schematic model for the 3D finite-difference time-domain (3D-FDTD) method using a software "RSoft-FullWAVE." A three-dimension system with periodic boundary conditions in the *x* and *y* directions, was used for a nanohole structure consisting of silica (*n*: 1.470 for wavelength 400 nm, *n*: 1.452 for wavelength 900 nm) and water (*n*: 1.33). A broadband (400–900 nm) plane wave source polarized along the *x*-axis or *y*-axis was incident from within the water region. Considering the unpolarized nature of the sunlight, the transmission spectrum is averaged among incident electric field polarized along *x*-axis and *y*-axis. The thickness of the silica layer varied from 130 to 250 nm to verify the effect of the thickness of the silica plate. The difference in transmittance between the silica plates with and without nanoholes was then examined with a plate thickness 150 nm.
